# Dynamics of the Force of Infection: Insights from *Echinococcus multilocularis* Infection in Foxes

**DOI:** 10.1371/journal.pntd.0002731

**Published:** 2014-03-20

**Authors:** Fraser I. Lewis, Belen Otero-Abad, Daniel Hegglin, Peter Deplazes, Paul R. Torgerson

**Affiliations:** 1 Section of Veterinary Epidemiology, University of Zürich, Zürich, Switzerland; 2 Institute of Parasitology, University of Zürich, Zürich, Switzerland; Swiss Tropical and Public Health Institute, Switzerland

## Abstract

Characterizing the force of infection (FOI) is an essential part of planning cost effective control strategies for zoonotic diseases. *Echinococcus multilocularis* is the causative agent of alveolar echinococcosis in humans, a serious disease with a high fatality rate and an increasing global spread. Red foxes are high prevalence hosts of *E. multilocularis*. Through a mathematical modelling approach, using field data collected from in and around the city of Zurich, Switzerland, we find compelling evidence that the FOI is periodic with highly variable amplitude, and, while this amplitude is similar across habitat types, the mean FOI differs markedly between urban and periurban habitats suggesting a considerable risk differential. The FOI, during an annual cycle, ranges from (0.1,0.8) insults (95% CI) in urban habitat in the summer to (9.4, 9.7) (95% CI) in periurban (rural) habitat in winter. Such large temporal and spatial variations in FOI suggest that control strategies are optimal when tailored to local FOI dynamics.

## Author Summary

Human alveolar echinococcosis (AE) is caused by the fox tapeworm *E. multilocularis* and has a high fatality rate if untreated. The frequency of the tapeworm in foxes can be reduced through the regular distribution of anthelmintic baits and thus decrease the risk of zoonotic transmission. Here, we estimate the force of infection to foxes using a mathematical model and data from necropsied foxes. The results suggest that the frequency of anthelmintic baiting of foxes can be optimised to local variations in transmission that depend upon season and type of fox habitat.

## Introduction

The force of infection (FOI) is a crucial epidemiological parameter and characterizing its dynamics is an essential part of planning cost effective control strategies for infectious diseases [1]. Mechanistically, disease intervention strategies are typically targeted at decreasing the per capita infection rate. If successful, this will then cause a decrease in observed prevalence. As such, quantification of the FOI provides a key measure of efficacy when assessing or comparing interventions [2]. The FOI can be extremely difficult to estimate directly, i.e. observationally, in wildlife populations. Even in human populations this is not without considerable challenges, and requires accurate longitudinal monitoring of the target population in order to capture all new infections which arise [3]. An alternative approach is to estimate the FOI indirectly, through access to prevalence data, in conjunction with either an explicit mathematical model describing the disease transmission processes, or else some assumed disease risk function [4], [5].

Foxes are typical definitive hosts for the parasite *Echinococcus multilocularis*, with different rodent species being the primary intermediate host in which the alveolar hydatid cysts grow. In humans, which are aberrant hosts, this parasite causes the important emerging zoonosis alveolar echinococcosis (AE). This is a serious disease with a high fatality rate in the absence of appropriate treatment [6]. In Europe there have been increasing numbers of AE cases reported in the Baltics [7], Poland [8], Austria [9] and in Switzerland [10]: the latter associated with an increase in fox populations. The disease is also emergent in central Asia with a huge increase in the numbers of human cases in Kyrgyzstan recorded in recent years [11]. This disease also has a considerable impact on human health in Western China, particularly on the Tibetan plateau [12]. Alveolar echinococcosis is also an emerging public health concern in North America due, at least in part, to the increasing urbanization of wild canids [13]. Red foxes (*Vulpes vulpes*) are high prevalence hosts of *E. multilocularis* [14], where zoonotic transmission may occur through environmental contamination [15] or through contaminated food [16]. In addition, dogs are susceptible definitive hosts [17] and may be very important for transmission to humans where prevalences in dogs are high, such as in China [18] or central Asia [19]. In Europe, dogs are low pravalence hosts [20], but nevertheless may pose a high risk of introducing the parasite in non endemic countries such as the UK if appropriate treatment is not given when dogs enter the country [21].

In terms of potential control measures for reducing the risk of AE, a number of different studies have investigated anthelmintic baiting in foxes [22]. The impact of such approaches on reducing prevalence appears to strongly depend on the specific design used, in relation to how the baits are delivered and choices of location, and frequency. In Switzerland, year round monthly anthelmintic baiting is an effective control measure in foxes [22]. The *E. multilocularis* transmission cycle is, however, dynamically highly complex with many known temporal-spatial heterogeneities (for example [23]). Adopting, therefore, a baiting strategy in close concordance with FOI dynamics could optimize existing intervention strategies. In planning such intervention studies knowledge of the dynamics and magnitude of the FOI can be invaluable, as this potentially allows the frequency of baiting to be tailored to the changing levels of exposure throughout time and across space. This may enable considerable cost saving, as opposed to, for example, monthly all year round baiting across all habitat types.

In Switzerland it has been shown that there are considerable differences in the spatial and seasonal distribution of the prevalence of *E. multilocularis* in definitive hosts [14], [15] and intermediate hosts [23]. These studies indicated that 129 of 857 *Arvicola terrestris* were infected of which 12 harboured protocolices. Ten of these animals had between 61 and 452,000 protoscolices. Seasonal patterns of infection in intermediate hosts were seen with highest prevalences seen in over-wintered animals. Thus seasonal anthelmintic treatment of foxes, with a focus on the autumn and winter months, is likely to be a more efficient strategy in reducing the parasite biomass [23]. Likewise although fox densities are highest in urban settings, they consume fewer rodents and have a greater reliance on anthropomorphic food supplies compared to rural foxes [24], which is likely to significantly affect transmission dynamics on a spatial scale. Consequently, the intensity of intervention strategies could also be tailored to exploit these spatial differences. Such differences in prevalences clearly indicate that relative differences in the FOI exist between rural and urban areas, and between winter and summer seasons.

We develop a statistically robust quantitative characterization of the FOI for *E.multilocularis* in foxes to address three specific research questions: i) firstly, is the FOI constant or dynamic (with age of the host), and what is its value accounting for complexities such as statistical uncertainty; ii) secondly, how much does FOI vary quantitatively with habitat type, in particular between more or less urbanized regions; iii) and thirdly how much does the FOI of infection vary quantitatively on a temporal basis between winter and summer seasons.

## Methods

The key methodological aspect of this study is to identify an epidemiologically useful disease transmission model for *E.multilocularis* in foxes. A model whose structure can be objectively justified, and whose parameter estimates provide tangible insight into the key infection processes. Three sources of information are available to support model development: i) prevalence data from a previously presented observational study [24]; ii) approximate estimates as to likely survival times of *E. multilocularis* in foxes from experimental work [17]; and iii) existing transmission modelling frameworks for *Echinococcus granulosus* transmission in sheep and dogs [25]. Using [25] as a starting point, we identify a process model whose structure is an optimal fit to the prevalence data from [24], whilst making use of the parameter estimates from [17] as expert knowledge. Following [25] we utilize ordinary differential equations (ODEs) to describe the transmission dynamics, and to take advantage of prior knowledge from [17] we adopt a Bayesian paradigm [26] for all model fitting and statistical inference.

### Study data

The data to which we fit our transmission models is an extension of that previously described in [14] and [24], and includes only samples taken prior to the anthelmintic baiting intervention described in [27]. Samples were collected from in or around the city of Zurich in Switzerland. Three key variables were utilized: i) presence (absence) of *E.multilocularis* infection based on necropsy (details given in [14], [24]); ii) the age of each fox, and following previous studies, and as described in [14], cubs were assumed to be born on 1st April and age determination of foxes sampled after 1st July was done via examination of teeth (details given in [14]). Along with the date of death (which is known as these animals were culled by hunters) and the weight at death, each animal's approximate age in years and days was estimated. The final variable utilized was habitat type, where this comprised three zones reflecting differing degrees of urbanization: urban; border; and periurban. The characteristics of these are described in detail in [27]. The urban zone comprises of mostly residential dwellings with relatively few green spaces, the periurban zone is rural comprising of forests, fields, pastures, and meadows. The border zone separates urban from rural, and was defined as extending 250 meters from the edge of the urban area and into 250 meters of the periurban surroundings. The border zone includes largely residential areas, public spaces, allotments and pastures. The data used in the study is in the Supporting Information Data S1. Out of the 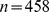 foxes aged three years or less in the study data, 160 were sampled in the periurban zone, 167 in the border zone and 131 in the urban zone. The overall observed prevalence across all 458 animals was 42.1%, within the periurban, border and urban zones this was 65.6%, 38.9% and 17.6% respectively. The median age across these 458 animals was 0.80 years. In the periurban, border and urban zones the median respective ages were 0.87, 0.77 and 0.59 years.

### Disease transmission model

**Figure 1:**
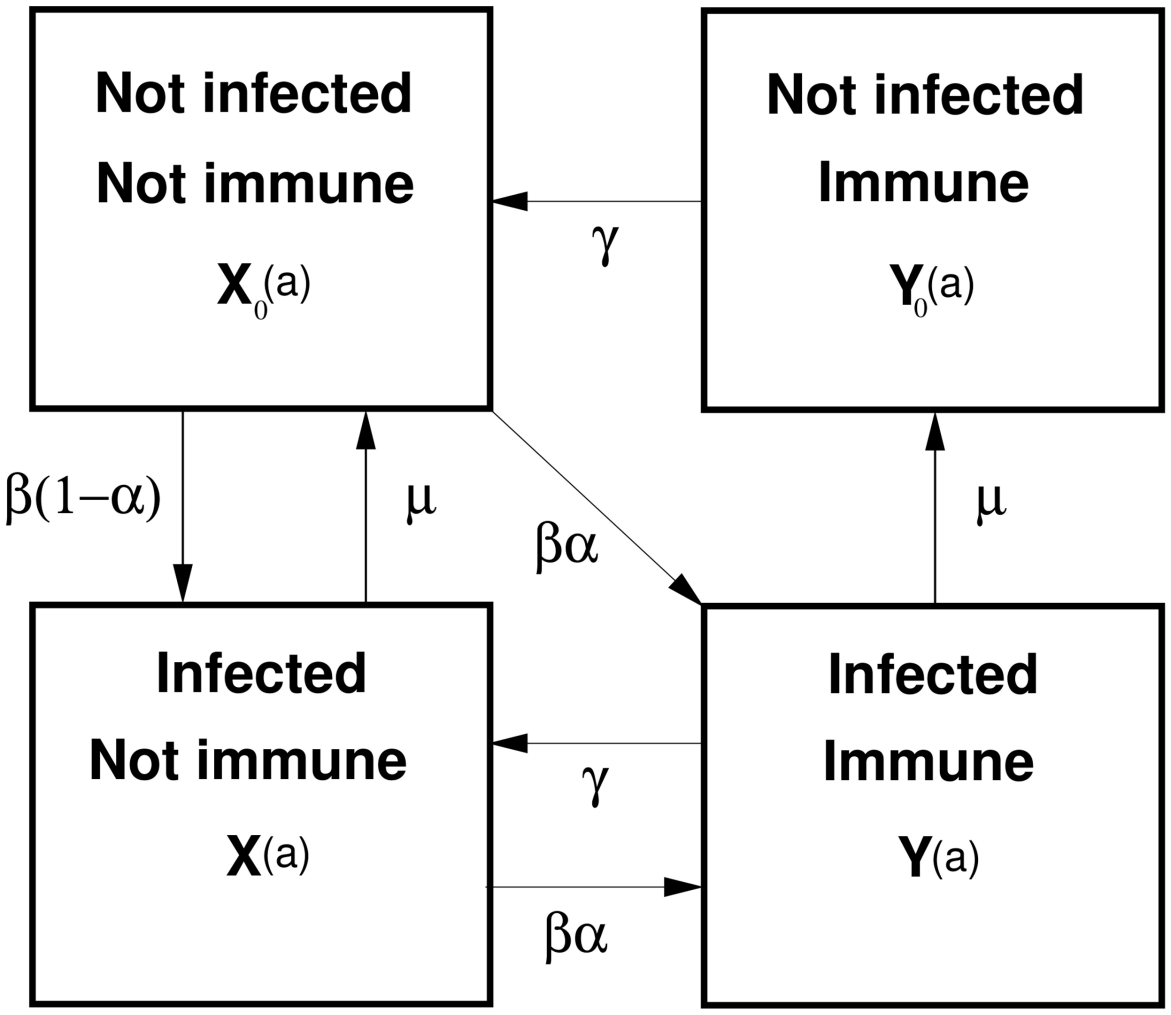
Transmission model for *E.multilocularis* in foxes. State variables are: 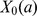, 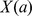, 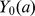 and 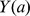, where 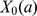 represents the proportion of hosts (foxes) which are not infected and not immune at age 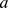, the other state variables are similarly defined. Parameter 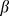 denotes the infection pressure (force of infection), measured in insults (exposures) per year; 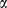 is the probability of immunity on exposure; 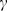 is the rate of loss of host immunity; 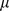 is the parasite death rate.

The most general form of hypothesized transmission model we consider for *E. multilocularis* is given in Figure 1. The structure of this model is based on initial work by [25] which has provided a basis for many subsequent disease modelling studies involving in *E. granulosus* and *E. multilocularis*, (e.g. [5],[28]). Figure 1 depicts an intuitively reasonable representation of the possible disease states and flows between them based on current known biology of *E.multilocularis* in foxes. The model dynamics here are over age of the host (foxes), as is typical when modelling *E. multilocularis* or *E.granulosus*. We assume a fully susceptible population at birth, i.e. no vertical transmission and therefore 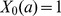. This dynamic system can be described in a series of ordinary differential equations (ODEs).

State variables are 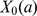, 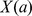, 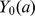 and 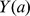, where 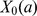 represents the proportion of hosts which are not infected and not immune at age 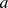, 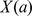 is the proportion of hosts which are infected and not immune at age 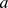. Variables 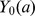 and 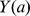 are defined similarly but for cohorts –not infected and immune} and –infected and immune} respectively. The following system of ordinary differential equations defines the dynamics over age of this system:
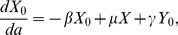


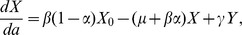


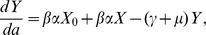


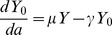
with initial conditions: 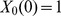, 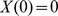, 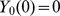 and 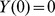. Parameter 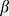 denotes infection pressure (force of infection - FOI), measured in insults (exposures) per year; 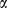 is the probability of immunity on exposure; 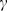 is the duration of host immunity; 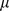 is the parasite death rate. Note that to simplify the notation we have suppressed any explicit dependency of the parameters on age, e.g. 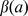 where FOI is dependent upon age, but such dependencies are considered during the model selection process making this potentially an inhomogeneous ODE system.

### Model fitting and statistical analyses

The observed data comprise of randomly sampled binary observations each denoting whether a fox was infected (not infected). This gives a sampling model comprising of Bernoulli trials where the likelihood function for 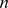 observations is 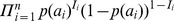, where 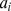 is the age of the 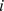
th fox in the data, 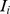 is an indicator variable where 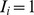 if the 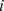
th fox is infected and 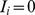 otherwise, and 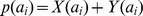 is the prevalence in foxes of age 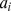. The ODE transmission model provides 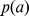 which will generally be some unknown function of the epidemiological parameters of interest, 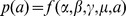 where (Figure 1): 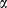 is the probability of immunity on exposure; 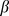 the force of infection (measured in insults per unit time); 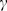 the rate of loss of immunity; and 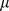 the parasite death rate. It is not necessary to know function 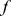 explicitly, all that is required is that for any given values of 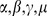, along with appropriate initial conditions for state variables 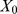, 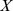, 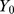, 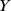, an estimate for 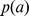 for any suitable value of 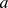 can be computed. This is readily possible using standard numerical techniques for solving ODEs (e.g. [29]). The likelihood function (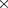 parameter priors as we are using Bayesian inference) can therefore be evaluated, and thus the key unknown epidemiological parameters of interest such as 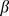 can be estimated from the study data —conditional on the chosen form of ODE model.

Gaussian distributed prior distributions for parameters 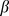 and 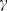 were used, where these were each implemented within a log link function. For the probability parameter 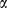, a logit link function was used, again with a Gaussian prior distribution. Highly diffuse priors were used for all parameters except 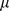, where these each had a mean of zero and standard deviation of 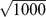. In effect, this introduces no prior biological knowledge into the estimation of these parameters. For 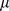, a Gaussian prior (again on a log link) was used and chosen via expert opinion based on data presented in [17]. The latter study comprised of longitudinal observation of five foxes experimentally infected with *E. multilocularis*. The parasite burden in 80% (three of five) animals was very low at 90 days, suggesting an 80th percentile for the death rate of approximately 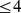 per year, in addition we consider that parasites in 50% of infected animals may survive to around 120 days (death rate 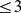 per year), with 2.5% possibly surviving beyond 150 days (death rate 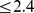 per year). A Gaussian distribution on a log link with a mean of 1.2 and standard deviation of 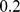, gives quantiles for 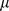 (on real scale) of approximately 2.24 (2.5%), 3.32 (50.0%) and 3.93 (80%) per year, which we choose as an informative prior for 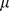. In addition we also examine a wider, but still highly informative prior, with a mean of 1.3 and standard deviation of 0.3 which has corresponding quantiles of 2.04 (2.5%), 3.67 (50.0%) and 4.72 (80%) per year. Sensitivity to prior assumptions is a crucial aspect of Bayesian inference, so we also present modelling results which use the same highly diffuse (uninformative) prior for 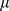 as for 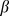 and 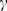.

Bayesian model selection — used to identify an optimal ODE transmission model — was performed using the marginal likelihood goodness of fit metric. This is equivalent to comparing Bayes factors between two models when each has an equal a priori probability of being the preferred model. The marginal likelihood is generally more difficult to compute than other commonly used metrics, such as the Bayesian Information Criterion (BIC) or Deviance Information Criterion (DIC), but is the standard and preferred theoretical choice in Bayesian inference [26], [30]. This metric allows Bayesian model selection to be interpreted as simply an extension of maximum likelihood model selection, where evidence (i.e. statistical support) for any given model is that obtained by multiplying the best fit likelihood by the “Occam factor”, so-named as this metric has been shown to be conceptually consistent with Occam's Razor (as explained in [30]). The marginal likelihood was computed using Laplace approximations, a standard numerical technique in statistical inference [31], [32]. These were also used to estimate posterior distributions for the epidemiological parameters. All numerics were implemented in R [33] using a number of well tested internal functions borrowed from the R abn library [34]. See Supporting Information Text S1 for technical details. An approximate guide for the size of differences in marginal likelihoods which may be considered notable is given in Table 2.1 page 27 in [26]. Using the terminology from [26], a difference of 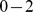 is suggested as weak support for the model with higher marginal likelihood, 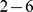 is support, 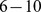 is strong evidence and greater than 10 very strong evidence.

## Results

We first present a brief exploration of the observed prevalence data by age. This is prudent as it may suggest refinements in the parametrization of the process models under consideration. Next we compare the goodness of fit of a range of models with different biological assumptions, for example whether the observed data support the presence of immunity, and if so, whether this is lifelong or transient. We then quantify the key epidemiological parameters in our chosen model, in particular the FOI, 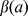. Heterogeneity is then introduced into this model by allowing the force of infection to differ across one or more of the three different habitat types, where further model selection is used to identify a preferred heterogeneous model. Our results conclude with a comparison of FOI estimates across the different habitat zones.

### Exploratory analyses by age

**Figure 2:**
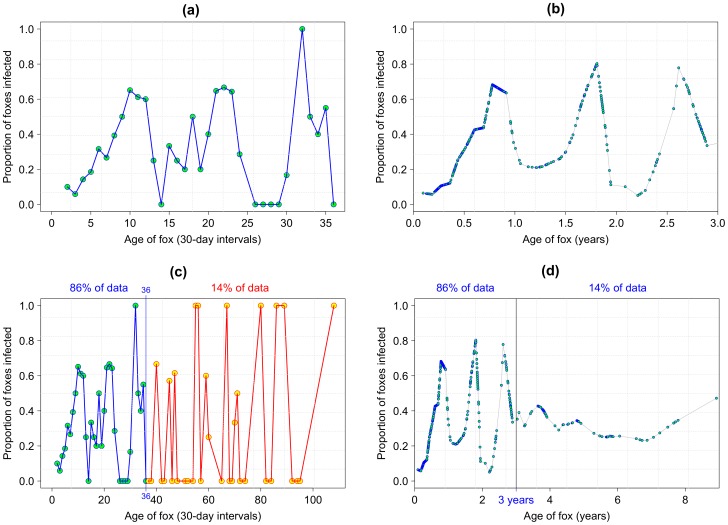
Exploratory analyses. Panel (a) shows observed prevalence across age groups of 30-days blocks up to age 36 months (where 1 month = 30 days). Panel (b) shows smoothed prevalence using a locally weighted regression smoother (lowess() in R) applied to the 0/1 observation for all individuals aged less than 3 years. Panel (c) shows observed prevalence across age groups of 30-days blocks for all ages (maximum 108 months where again one month = 30 days). Panel (d) shows the smoother applied to data of all ages.

Exploratory analyses of the observed prevalence data is illustrated in Figure 2. Choosing a smoothing parameter of f = 0.072 in (lowess() in R) gives smoothed data which appear relatively consistent with the observed data in Figure 2 (a), and provides a more refined visualization of the data rather than in 30-day blocks. Figure 2 (a) and 2 (b) suggest that it may be appropriate to consider the inclusion of periodicity into one or more of the epidemiological parameters in our transmission model.This suggests that for our model to adequately capture the gross dynamic features of disease transmission we should consider both age independent FOI, 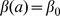, and also FOI parametrized as a function of age, 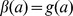, with 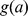 as some polynomial or periodic function. It is clear from Figure 2 (c) that there appears very little identifiable dynamic structure after 36 months, which is perhaps unsurprising given this only comprises some 14% on observations, and thus very sparse sampling at these older ages.This is consistent with life expectancy estimates for foxes which suggest that only a small proportion of foxes survive beyond 2–3 years years in the wild [35]. As foxes aged less than three years present the vast majority of zoonotic risk, combined with foxes of older ages being sampled very sparsely in the data, subsequent analyses focus on foxes less than three years of age. For completeness some modelling results are also presented considering all ages. Figure 2 (d) shows the smoother applied to data of all ages.

### Determining a parsimonious transmission model

A range of transmission models of increasing complexity were fitted to the observed data (Table 1) with separate results shown for the two informative priors for 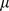. See Supporting Information Text S2 for results using an uninformative prior for 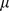, and Supporting Information Text S3 for the equivalent of Table 1 but for the models fitted to data from foxes of all ages. Estimates of the posterior modes for all the parameters in models presented in Table 1 can be found in Supporting Information Text S4.

**Table 1:** Model goodness of fits.

Model	Description	Prior for 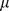	Log marginal likelihood
1-C	no immunity 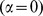 Constant FOI: 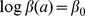	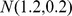 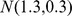	−305.3 ( 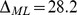 ) −304.3 ( 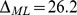 )
1-L	no immunity 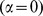 Linear FOI: 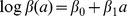	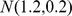 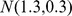	−309.3 ( 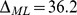 ) −308.9 ( 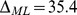 )
1-Q	no immunity 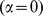 Quadratic FOI: 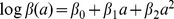	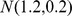 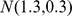	−308.1 ( 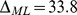 ) −308.3 ( 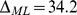 )
1-P	no immunity 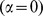 Periodic FOI: 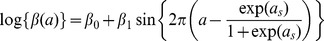	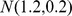 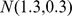	−291.3 ( 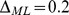 ) −291.2 ( 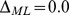 )
2	lifelong immunity 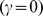 periodic FOI: 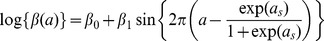	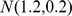 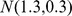	−294.3 ( 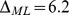 ) −294.6 ( 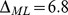 )
3	transient immunity 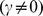 periodic FOI: 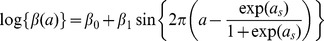	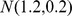 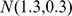	−294.2 ( 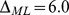 ) −296.0 ( 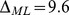 )

All parameters other than 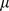 have diffuse priors as given in the text. The 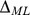 denotes twice the difference between the best log marginal likelihood and each of the other models.

### Evaluation of immunity

We commenced with a model comprising no immunity (Model 1-C), i.e. only state variables 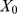 and 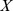, and constant FOI. This was followed by similar models but where the FOI was parametrized as a linear (1-L), quadratic (1-Q) and periodic (1-P) function of age, with the latter using a sinusoidal forcing term as is commonly used for diseases with periodic transmission rates (e.g. measles [36]). The particular form of sinusoidal function used was 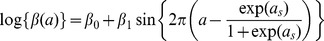. A log link function ensures that all estimates of 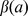 are positive, and also avoids the potentially complex task of having to specifying a proper (i.e. integrates to unity) joint parameter prior for 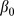, 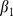 and 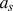 which would otherwise be required to ensure that the posterior distribution for 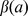 was positive. This parametric form of 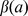 has a period of one year, with (on a log scale) 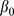 denoting the lifetime average (or baseline) FOI, 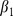 the amplitude beyond the lifetime average. The term 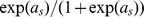 is to allow, if necessary, a time shift compared with the standard sinusoidal function. A logit link function is used here as we are only interested in time shifts in the interval [0,1]. Parameters 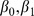 and 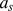 each have diffuse Gaussian priors with means of zero and standard deviations of 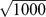.

From Table 1 is it clear that periodic infection pressure is strongly supported over the other forms. Retaining periodic infection pressure, we next consider models with a more complex cohort structure comprising of all four state variables 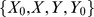, allowing for the presence of lifelong immunity (Model 2), and transient immunity (Model 3 and the “full” model in Figure 1). It is again apparent from Table 1 that the observed data are less supportive of these two more complex models, and hence there is little evidence in the data for the presence of immunity.

Based purely on the goodness of fit results in Table 1 our preferred model is Model 1-P. The next more complex best fitting model was Model 2. These two models cross a rather large biological divide — no immunity verses lifelong immunity. To provide additional empirical justification for choosing Model 1-P over Model 2 we briefly examine the magnitude of the parameters in the latter model using the posterior modes (which are estimated as part of the marginal likelihood computation). In Model 2, using the prior for 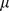 with mean of 1.2, we have a logit for 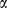 of −5.3 giving an approximate probability of becoming immune per exposure of 0.005. Posterior mode estimates for the FOI in this model, 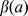, gives an (approximate) average lifetime number of exposures, 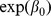, of 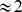 per year. Based on the observed prevalence data, then suppose that 86% of animals have a lifetime of at most three years and the remaining 14% live for a full nine years. Then, in a population of 100 animals these parameters give a total of 768 exposures for all animals over their entire lifetime. For 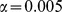 this then gives, on average, at most only four animals becoming immune during the entire lifetime of the population. This is a very fine scale population change, and it is therefore of little surprise that, statistically, the empirical data are not supportive of the presence of immunity.

### Quantification of force of infection

Having arrived at a preferred transmission model we now use this to provide the first of our main results: quantification of the FOI, i.e. 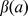. Of most interest here are the baseline and amplitude parameters 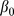 and 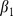, specifically we wish to estimate the joint marginal posterior distribution for these two parameters and then examine the range of values for the FOI which arise when 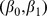 are within their joint 95% posterior confidence interval (to account for sampling uncertainty). It would be possible to consider a joint density comprising of all three parameters in 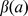; 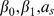. It is, however, difficult to visualize such a density (with four dimensions - three parameters plus the density estimate), and as epidemiological interest is focused on 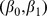 we therefore marginalize out 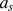 and 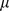 giving a joint posterior density for 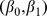. Note that this distribution, therefore, also incorporates the statistical uncertainty in 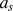 and 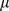 (i.e. the latter are not simply fixed at constant values).

Before computing the joint marginal density for 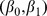 we first summarize 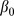, 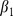, 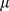 and 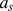 through their marginal posterior 95% confidence intervals (Supporting Information Text S5 provides full marginal posterior densities). Using the informative prior for 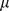 with mean = 1.2 and sd = 0.2 gives (on the real scale) 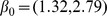, 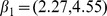, 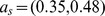 and 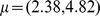, with approximate medians of 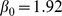, 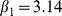, 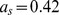; and 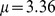. The corresponding estimates when using the informative prior for 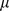 with mean = 1.3 and sd = 0.3 are 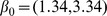, 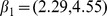, 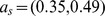 and 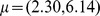, with approximate medians of 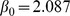, 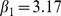, 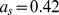; and 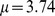. Using the diffuse prior for 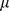 gives 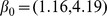, 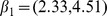, 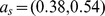 and 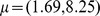, with approximate medians of 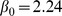, 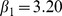, 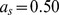; and 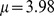.

**Figure 3:**
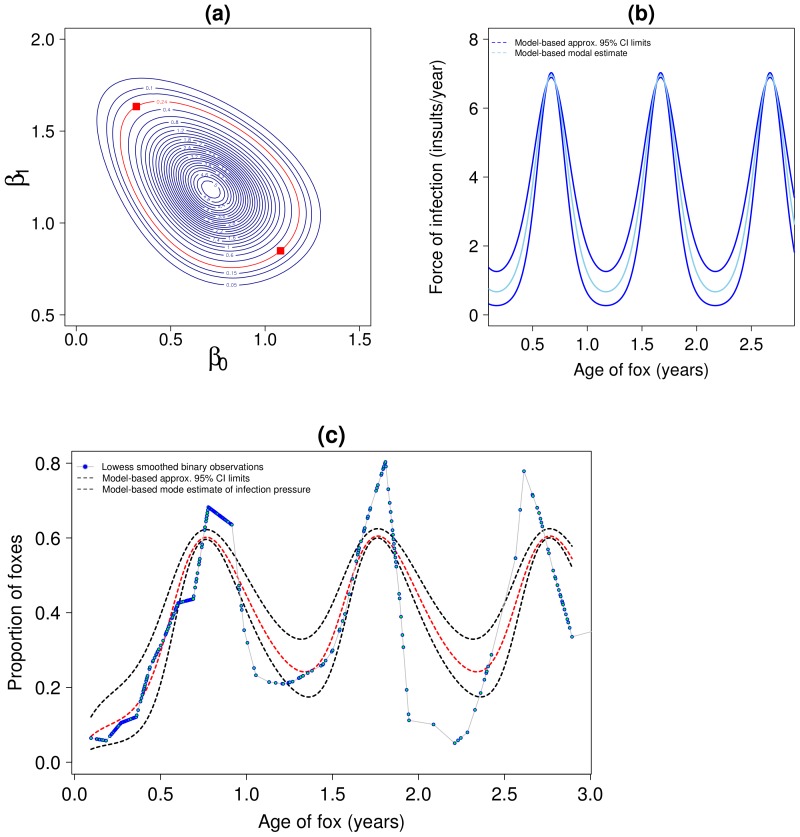
Transmission Model 1-P. Panel (a): joint marginal posterior density for 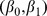 on log scale. The red contour is the 95% limit and the two points marked are those used to produce approx. 95% confidence intervals in panels b and c. Panel (b): dynamics of force of infection by age, 95% CI is for the mean force of infection at age 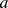. Panel (c): Smoothed observed prevalence and prevalence predicted by Model 1-P, 95% CI are for the mean prevalence at age 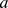. All results use the informative prior for 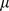 with mean = 1.2 and sd = 0.2.

A contour plot of the joint marginal posterior density for 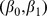, Figure 3 panel a, clearly shows strong dependency between 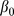 and 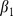 — when one is lower the other is higher and vice-versa. This demonstrates why it is more intuitively reasonably to consider these parameters jointly. To visualize the statistical uncertainly in our estimate of FOI over age we choose two points 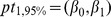 and 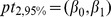, which lie on the contour defining the 95% region for this two-dimensional density. We then solve the ODE model for these sets of parameter estimates (the other two parameters are set to their modal values). These two “extreme” sets of parameters provide an approximate 95% confidence interval for the mean force of infection over age (Figure 3 panel b), and similarly the mean prevalence (Figure 3 panel c). We estimate the (mean) minimum FOI during an annual population cycle as 0.27 to 1.27 insults (with 95% confidence), and rising to a maximum of between 6.87 and 7.05 insults (with 95% confidence).

### Comparison between urban and rural habitats

The summary statistics suggest that there may be a difference between the prevalence of *E.multilocularis* in populations of foxes within the different habitat types. To provide a measure of statistical rigour to these observations we fit Model 1-P to these data, where now heterogeneity is introduced into 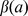 to allow the force of infection to vary across each of the different zones. If the inclusion of such heterogeneity improves the model goodness of fit then that provides formal statistical evidence of a different in FOI between habitats.

We consider two versions of Model 1-P, Model 1-P^0^ and Model 1-P^01^. The first allows the baseline force of infection, 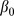, to vary with zone and assumes the amplitude 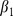 is homogeneous across all zones. The second model allows both 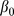 and 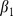 to vary within each habitat zone. For simplicity, the period shift 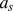 and parasite death rate 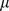 are assumed homogeneous over all three zones. Model 1-P^0^ has a goodness of fit of −285.4, with Model 1-P^01^ having −292.6. This is strong evidence that: i) there is a difference in baseline force of infection between different habitat zones; ii) there is no evidence of any difference in periodic amplitude between the different habitats. We use, therefore, Model 1-P^0^ to quantify differences in FOI across habitat.

Following a similar approach as for our analyses of Model 1-P, we derive approximate confidence intervals for the force of infection using the joint marginal posterior densities for 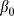 and 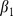, where this time we have three, two dimensional distributions, 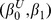, 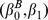, 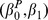 for 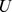 urban, 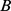 border and 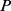 periurban. First we summarize 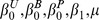 and 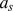 through their marginal posterior 95% confidence intervals (Supporting Information Text S6 provides full marginals posterior densities). Using the informative prior for 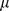 with mean = 1.2 and sd = 0.2 gives (on the real scale) 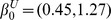, 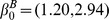, 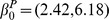, 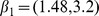, 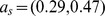 and 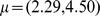, with approximate medians of 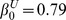, 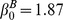, 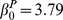, 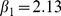, 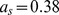 and 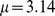. It is clear that the marginal densities in the urban and periurban habitats do not overlap at the 5% significance level. Supporting Information Text S7 provides a comparison of the modal estimates of prevalence over age in each of the three habitat types.

**Figure 4:**
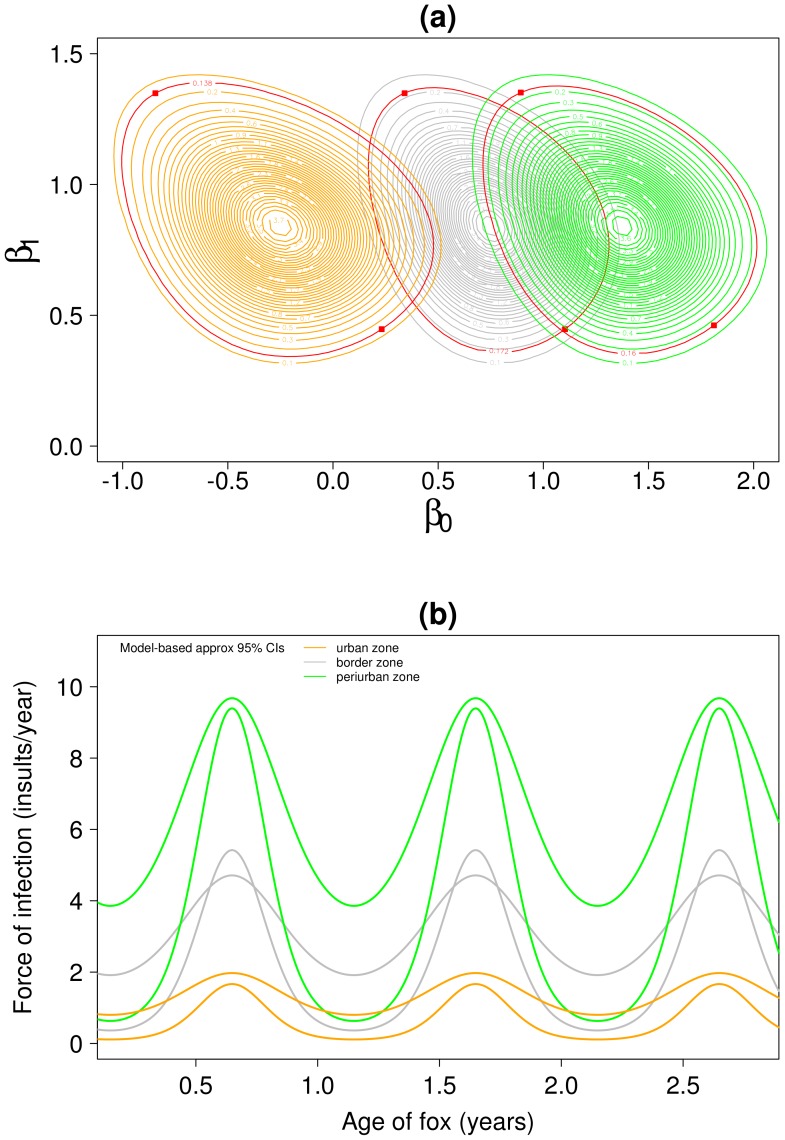
Heterogeneous habitat transmission Model 1-P^0^. Panel (a): joint marginal posterior densities for 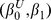, 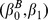, 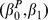 on log scale. The red contour is the 95% limit and the two points marked are those used to produce approx. 95% confidence intervals in panel b. Panel (b): dynamics of force of infection by age, approx 95% CI is for the mean force of infection at age 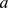 (see main text for explanation of why these lines cross). All results use the informative prior for 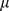 with mean = 1.2 and sd = 0.2.

Finally we consider the statistical uncertainty in our FOI estimates over age within each habitat type. Figure 4 panel a is similar to Figure 3 panel a and shows the joint marginal posterior densities for 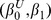, 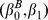, 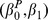. As for the one-dimensional marginal estimates of 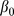 in each habitat, it is very clear that the FOI baseline is statistically different between the urban and periurban zones i.e. the 95% contours do not overlap. The FOI in the border zone is indistinguishable from that in either the periurban or rural zones. We repeat the same approach to estimate approximate 95% confidence intervals for the FOI within each habitat as for the homogeneous habitat model (Model 1-P), this is shown in Figure 4 panel b. These uncertainty limits are clearly rather more approximate here than for those in Model 1-P — as can be seen by the fact that the urban and periurban trajectories overlap slightly, while they are clearly very distinct at the 95% contours in Figure 4 panel a. The limits for the border habitat also cross each other. This behavior is not entirely unexpected in that we are collapsing a six dimensional posterior probability distribution (comprising of all the parameters in Model 1-P^0^) into effectively only two dimensions. This gives joint statistical estimates which are far more manageable, but as we see here, does makes the resulting confidence limit estimates rather approximate. We estimate with approximate 95% confidence that the (mean) minimum FOI during an annual cycle in the urban habitat is 0.1 to 0.8 insults, rising to a maximum of between 1.6 and 2.0 insults. For the periurban habitat we have minimum and maximum force of infections of 0.7 to 3.9 insults and 9.35 to 9.7 insults respectively. Despite these minor statistical discrepancies in relation to the differing comparisons of confidence limits, the overall result is very clear: there is a large difference in FOI during annual cycles in the urban and periurban habitats.

## Discussion

The FOI is a key parameter in models estimating the effectiveness and cost effectiveness of infectious disease prevention [37]. Using a simple —and empirically justified — mathematical model we have estimated the force of *E. multilocularis* infection in a fox population in Switzerland, and shown how much it quantitatively varies with season and geography, i.e. through time and across space.

There have been a number of trials aimed at reducing the prevalence of infection in foxes by distributing baits containing the anthelmintic praziquantel. Several studies, in Switzerland and in Germany, with baiting intervals of 12 times per year, resulted in a substantive decline in the numbers of foxes infected (reviewed in [22], [38], [39]). These studies typically resulted in a decrease in prevalence from 35% and 67% to between 1% and 6%. Provided most foxes are treated, this would be expected as the baiting interval is similar to the prepatent period of *E. multilocularis* in foxes and hence it should prevent transmission. Other baiting campaigns have used lower frequencies and have had variable results. For example in Germany a baiting frequency of 5 times per year resulted in a decrease in the prevalence in foxes of 32% (95% CIs 16–52) to 4% (95% CIs 2–7). Other studies with less frequent baiting intervals have not shown such a clear reduction. Our estimates and modelling methodology for computing the pre-intervention baseline FOI provides a rigorous framework which can be used to optimize baiting intervals, in order to trade off the need to reduce infection in foxes, and thus the potential for zoonotic transmission, and the cost of implementing such intervention programmes. Based on Swiss data we estimate that there is a high infection pressure in the winter months for non urban foxes of close to 10 infections per year (i.e. greater than 1 per month), baiting at monthly intervals would therefore be required. This conclusion is in accordance with the results of an epidemiological study on the intermediate hosts which showed most rodents become infected during the winter [23]. However, in the summer when the FOI is lowered to between 0.7 to 3.9 insults per year, then decreasing the baiting frequency to once every three months would be more appropriate. In addition, baiting frequency, at least in theory, could be further reduced in urban habitats where the FOI is between 0.1–0.8 and 1.6–2.0 insults per year. However in practice, this would be a challenge in Zürich as the spatial separation of such zones is as little as 500 meters. A decreased cost of baiting foxes increases the cost benefit as a similar reduction in the numbers of human AE cases would be expected to be achieved as earlier suggested [15] based on epidemiological data [23], [24]. Theoretical models [40], [41], have also suggested seasonal transmission of *E. multilocularis* in Japan. However, our model is also challenged with field data, where as the conclusions of previous models are based on simulations. In addition, our model does not depend upon parameters from the intermediate host and therefore should be applicable for FOI calcualtions in any area where suitable prevalence data from foxes is available.

Our estimates of FOI are dependent on the estimate of the life expectancy of the infection in the definitive host. Experimental infections of foxes indicate that parasites can survive in foxes beyond 90 days [17], although most parasites are lost earlier. This model is based on the presence or absence of parasites, with even a single parasite being found in a fox defining the fox as infected. Therefore an estimated life expectancy of 120 days was used in the model as being a reasonable period extrapolating from the data of [17]. By which half of foxes might be estimated to be free of parasites. If the life expectancy is less then the FOI will be higher than reported here. The corollary is also true. A longer life expectancy would result in a lower FOI. It is possible that low worm burdens in foxes could persist for some considerable time as all foxes in the experimental study by Kapel and others [17] remained infected at 90 days, albeit with low burdens. However, if this were the case, decreasing baiting frequency in the summer months and in urban areas, as suggested would still be effective in lowering the parasite biomass, as the numbers of infections per year would be lower than calculated here. However, as infection is highly overdispersed only a few infected foxes will be responsible for most of the transmission. Using a non zero threshold worm burden for foxes that are relevant to transmission could give important information with regard to the FOI in heavily infected foxes. An alternative approach, in a future study, using abundance data may help clarify this issue. An obvious related key question is quantifying the transmission probability from environmental contamination, e.g. via the distribution of fox faeces, to human infection.

To finish, a brief comment on the basic reproduction ratio (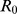), arguably the most important epidemiological parameter in any disease system, although it is not without its critics [42]. Robust estimation of 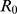 is often difficult, especially with parasites with complex life cycles. Roberts [43] described how 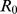 could be estimated if prevalence data from foxes and small mammal intermediate hosts were available together, along with a number of assumptions regarding various transmission parameters. However, when it is difficult to estimate 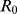, estimates of FOI become highly relevant [37]. We have shown that with a relatively simple transmission model empirically justified from study data, an estimate of the FOI can be made, and how this can be practically applied for optimizing the interval of baiting to lower the prevalence of *E. multilocularis* in foxes.

## Supporting Information

Data S1 **File containing original data.**
(XLS)

Text S1 **Estimating the marginal likelihood.**
(PDF)

Text S2
**Results using an uniformative prior for** 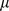 **.**
(PDF)

Text S3 **Modeling results for foxes of all ages.**
(PDF)

Text S4 **Estimates of the posterior modes for all the parameters in models presented in** **Table 1** **.**
(PDF)

Text S5
**Full marginal posterior densities for model 1-P for the parameters** 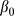 **,** 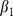 **,** 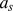 **and** 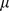 **using the informative prior** 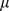 **with mean = 1.2 and s.d. = 0.2.**
(PDF)

Text S6
**Full marginal Posterior densities for model** 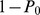 **for the parameters** 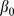 **,** 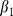 **,** 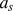 **and** 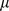 **using the informative prior** 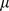 **with mean = 1.2 and s.d. = 0.2.**
(PDF)

Text S7 **Model prevalence estimates by habitat using model 1-P^0^.**
(PDF)
