# Supplementary material for: Dynamics of the Force of Infection: Insights from Echinococcus multilocularis Infection in Foxes
Source: PLoS Negl Trop Dis. 2014 Mar 20;8(3):e2731. doi: 10.1371/journal.pntd.0002731 (PMC3961194; doi:10.1371/journal.pntd.0002731)
Supplement: Text S1 — Estimating the marginal likelihood. (PDF) [file pntd.0002731.s002.pdf]

Supporting Information Text S1

Estimating the Marginal Likelihood

The marginal likelihood was estimated using the standard Laplace analytical approximation. Numerically, this was somewhat involved in order to ensure reliable estimates due to the need to use finite differencing rather than analytical derivatives. The computational code was written in C and called from within R using the R API. Extensive use was made of both the GNU Scientific Library (http://www.gnu.org/software/gsl/) and also some of R's internal optimization functions. The likelihood $\times$ prior function was optimized using R's L-BFGS-B (called internally from C), where at each step in the optimization the given system of ordinary differential equations (ODE) in the likelihood function was solved numerically, using GSL's ODE solver functionality with adaptive step-size and error controlling routines (in particular the explicit embedded Runge-Kutta Prince-Dormand (8, 9) method was used). The gradient function in the L-BFGS-B was provided via using GSL's adaptive finite difference routines. The Hessian estimate in the Laplace approximation was computed using finite differencing (again using GSL's routines) but where this was first nested inside a one dimensional minimiser in order to determine the initial step size value (provided to the GSL routines) in the finite difference approximation which resulted in the smallest (absolute) error between the estimate of the Hessian using a five point difference rule and a three point difference rule. The step size optimization was performed using GSL's Brent minimization algorithm. Once the optimal step size had been determined then the final, most robust value of the Hessian was determined. This rather lengthy approach was used as Hessian estimates using finite differencing can be rather sensitive to the step size used (or in this case the initial step size guess suggested to the adaptive GSL routines). This general approach is what is used in the R abn library and has been tested for robustness on a number of data sets.
