# Supplementary material for: Dynamics of the Force of Infection: Insights from Echinococcus multilocularis Infection in Foxes
Source: PLoS Negl Trop Dis. 2014 Mar 20;8(3):e2731. doi: 10.1371/journal.pntd.0002731 (PMC3961194; doi:10.1371/journal.pntd.0002731)
Supplement: Text S2 — Results using an uniformative prior for . (PDF) [file pntd.0002731.s003.pdf]

# Supporting Information Text S2

## Results using an uniformative prior for $\mu$

This is similar to Table 1 in the main manuscript but for the uninformative prior for $\mu$.

| Model | Description | Prior for $\mu$ | Log marginal likelihood |
|---|---|---|---|
| 1-C | no immunity ($\alpha = 0$)<br>Constant FOI:<br>$\log \beta(a) = \beta_0$ | $N(0.0, \sqrt{1000})$ | -303.2 ($\Delta_{ML} = 16.4$) |
| 1-L | no immunity ($\alpha = 0$)<br>Linear FOI:<br>$\log \beta(a) = \beta_0 + \beta_1 a$ | $N(0.0, \sqrt{1000})$ | -306.4 ($\Delta_{ML} = 22.8$) |
| 1-Q | no immunity ($\alpha = 0$)<br>Quadratic FOI:<br>$\log \beta(a) = \beta_0 + \beta_1 a + \beta_2 a^2$ | $N(0.0, \sqrt{1000})$ | -300.3 ($\Delta_{ML} = 10.6$) |
| 1-P | no immunity ($\alpha = 0$)<br>Periodic FOI:<br>$\log\{\beta(a)\} = \beta_0 + \beta_1 \sin\left\{2\pi\left(a - \frac{\exp(a_s)}{1+\exp(a_s)}\right)\right\}$ | $N(0.0, \sqrt{1000})$ | -295.0 ($\Delta_{ML} = 0.0$) |
| 2 | lifelong immunity ($\gamma = 0$)<br>periodic FOI:<br>$\log\{\beta(a)\} = \beta_0 + \beta_1 \sin\left\{2\pi\left(a - \frac{\exp(a_s)}{1+\exp(a_s)}\right)\right\}$ | $N(0.0, \sqrt{1000})$ | -296.2 ($\Delta_{ML} = 2.4$) |
| 3 | transient immunity ($\gamma \neq 0$)<br>periodic FOI:<br>$\log\{\beta(a)\} = \beta_0 + \beta_1 \sin\left\{2\pi\left(a - \frac{\exp(a_s)}{1+\exp(a_s)}\right)\right\}$ | $N(0.0, \sqrt{1000})$ | -297.4 ($\Delta_{ML} = 4.8$) |

.
