# Supplementary material for: Dynamics of the Force of Infection: Insights from Echinococcus multilocularis Infection in Foxes
Source: PLoS Negl Trop Dis. 2014 Mar 20;8(3):e2731. doi: 10.1371/journal.pntd.0002731 (PMC3961194; doi:10.1371/journal.pntd.0002731)
Supplement: Text S3 — Modeling results for foxes of all ages. (PDF) [file pntd.0002731.s004.pdf]

Supporting Information Text S3

Modeling results for foxes of all ages

This is similar to Table 1 in the main manuscript but for the models fitting to the data from foxes of all ages.

Model	Description	Prior for μ	Log marginal likelihood
1-C	no immunity ($\alpha = 0$)<br>Constant FOI:<br>$\log \beta(a) = \beta_0$	$N(1.2, 0.2)$<br>$N(1.3, 0.3)$	-354.6 ($\Delta_{ML} = 22.8$)<br>-354.0 ($\Delta_{ML} = 21.6$)
1-L	no immunity ($\alpha = 0$)<br>Linear FOI:<br>$\log \beta(a) = \beta_0 + \beta_1 a$	$N(1.2, 0.2)$<br>$N(1.3, 0.3)$	-360.6 ($\Delta_{ML} = 34.8$)<br>-359.6 ($\Delta_{ML} = 32.8$)
1-Q	no immunity ($\alpha = 0$)<br>Quadratic FOI:<br>$\log \beta(a) = \beta_0 + \beta_1 a + \beta_2 a^2$	$N(1.2, 0.2)$<br>$N(1.3, 0.3)$	-365.5 ($\Delta_{ML} = 44.6$)<br>-365.1 ($\Delta_{ML} = 43.8$)
1-P	no immunity ($\alpha = 0$)<br>Periodic FOI:<br>$\log\{\beta(a)\} = \beta_0 + \beta_1 \sin\left\{2\pi \left(a - \frac{\exp(a_s)}{1+\exp(a_s)}\right)\right\}$	$N(1.2, 0.2)$<br>$N(1.3, 0.3)$	-343.3 ($\Delta_{ML} = 0.2$)<br>-343.2 ($\Delta_{ML} = 0.0$)
2	lifelong immunity ($\gamma = 0$)<br>periodic FOI:<br>$\log\{\beta(a)\} = \beta_0 + \beta_1 \sin\left\{2\pi \left(a - \frac{\exp(a_s)}{1+\exp(a_s)}\right)\right\}$	$N(1.2, 0.2)$<br>$N(1.3, 0.3)$	-344.6 ($\Delta_{ML} = 2.8$)<br>-344.8 ($\Delta_{ML} = 3.2$)
3	transient immunity ($\gamma \neq 0$)<br>periodic FOI:<br>$\log\{\beta(a)\} = \beta_0 + \beta_1 \sin\left\{2\pi \left(a - \frac{\exp(a_s)}{1+\exp(a_s)}\right)\right\}$	$N(1.2, 0.2)$<br>$N(1.3, 0.3)$	-345.7 ($\Delta_{ML} = 5.0$)<br>-349.3 ($\Delta_{ML} = 12.2$)
