# Supplementary material for: Dynamics of the Force of Infection: Insights from Echinococcus multilocularis Infection in Foxes
Source: PLoS Negl Trop Dis. 2014 Mar 20;8(3):e2731. doi: 10.1371/journal.pntd.0002731 (PMC3961194; doi:10.1371/journal.pntd.0002731)
Supplement: Text S4 — Estimates of the posterior modes for all the parameters in models presented in Table 1 . (PDF) [file pntd.0002731.s005.pdf]

Supporting Information Text S4

Estimates of the posterior modes for all the parameters in models presented in Table 1

The following table gives estimates for the parameter modes in each of the models presented in Table 1 in the main text. NA - not applicable.

Model	Prior for μ	Posterior modes
1-C	$N(1.2, 0.2)$	$\{\alpha, \beta_0, \mu, \gamma\} = \{0, 0.64, 0.79, \text{NA}\}$
	$N(1.3, 0.3)$	$\{\alpha, \beta_0, \mu, \gamma\} = \{0, 0.50, 0.57, \text{NA}\}$
1-L	$N(1.2, 0.2)$	$\{\alpha, \beta_0, \beta_1, \mu, \gamma\} = \{0, 0.55, 0.23, 0.92, \text{NA}\}$
	$N(1.3, 0.3)$	$\{\alpha, \beta_0, \beta_1, \mu, \gamma\} = \{0, 0.46, 0.18, 0.74, \text{NA}\}$
1-Q	$N(1.2, 0.2)$	$\{\alpha, \beta_0, \beta_1, \beta_2, \mu, \gamma\} = \{0, -0.02, 2.10, -0.70, 1.12, \text{NA}\}$
	$N(1.3, 0.3)$	$\{\alpha, \beta_0, \beta_1, \beta_2, \mu, \gamma\} = \{0, -0.02, 2.09, -0.70, 1.12, \text{NA}\}$
1-P	$N(1.2, 0.2)$	$\{\alpha, \beta_0, \beta_1, a_s, \mu, \gamma\} = \{0, 0.72, 1.19, -0.32, 1.28, \text{NA}\}$
	$N(1.3, 0.3)$	$\{\alpha, \beta_0, \beta_1, a_s, \mu, \gamma\} = \{0, 0.83, 1.19, -0.29, 1.42, \text{NA}\}$
2	$N(1.2, 0.2)$	$\{\alpha, \beta_0, \beta_1, a_s, \mu, \gamma\} = \{-5.30, 0.76, 1.19, -0.30, 1.30, 0\}$
	$N(1.3, 0.3)$	$\{\alpha, \beta_0, \beta_1, a_s, \mu, \gamma\} = \{-4.83, 0.83, 1.20, -0.27, 1.39, 0\}$
3	$N(1.2, 0.2)$	$\{\alpha, \beta_0, \beta_1, a_s, \mu, \gamma\} = \{-4.56, 0.72, 1.18, -0.32, 1.28, 2.72\}$
	$N(1.3, 0.3)$	$\{\alpha, \beta_0, \beta_1, a_s, \mu, \gamma\} = \{-6.32, 0.83, 1.19, -0.28, 1.43, 2.76\}$
