# Supplementary material for: Dynamics of the Force of Infection: Insights from Echinococcus multilocularis Infection in Foxes
Source: PLoS Negl Trop Dis. 2014 Mar 20;8(3):e2731. doi: 10.1371/journal.pntd.0002731 (PMC3961194; doi:10.1371/journal.pntd.0002731)
Supplement: Text S5 — Full marginal posterior densities for model 1-P for the parameters , , and using the informative prior with mean = 1.2 and s.d. = 0.2. (PDF) [file pntd.0002731.s006.pdf]

Supporting Information Text S5

Full marginal posterior densities for model 1-P

This section provides marginal posterior density estimates for all parameters in Model 1-P.

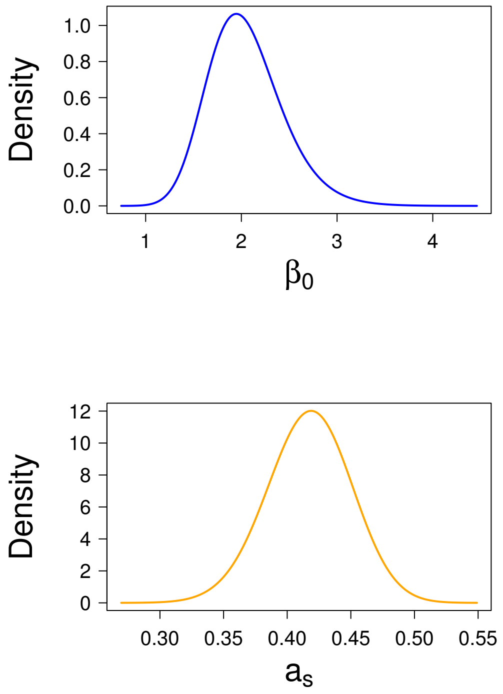

Marginal posterior densities for β_0, β_1, a_s and μ on the real scale using the informative prior for μ with mean=1.2 and sd=0.2
