# Supplementary material for: Dynamics of the Force of Infection: Insights from Echinococcus multilocularis Infection in Foxes
Source: PLoS Negl Trop Dis. 2014 Mar 20;8(3):e2731. doi: 10.1371/journal.pntd.0002731 (PMC3961194; doi:10.1371/journal.pntd.0002731)
Supplement: Text S6 — Full marginal Posterior densities for model for the parameters , , and using the informative prior with mean = 1.2 and s.d. = 0.2. (PDF) [file pntd.0002731.s007.pdf]

Supporting Information Text S6

Full marginal Posterior densities for model 1-P_0

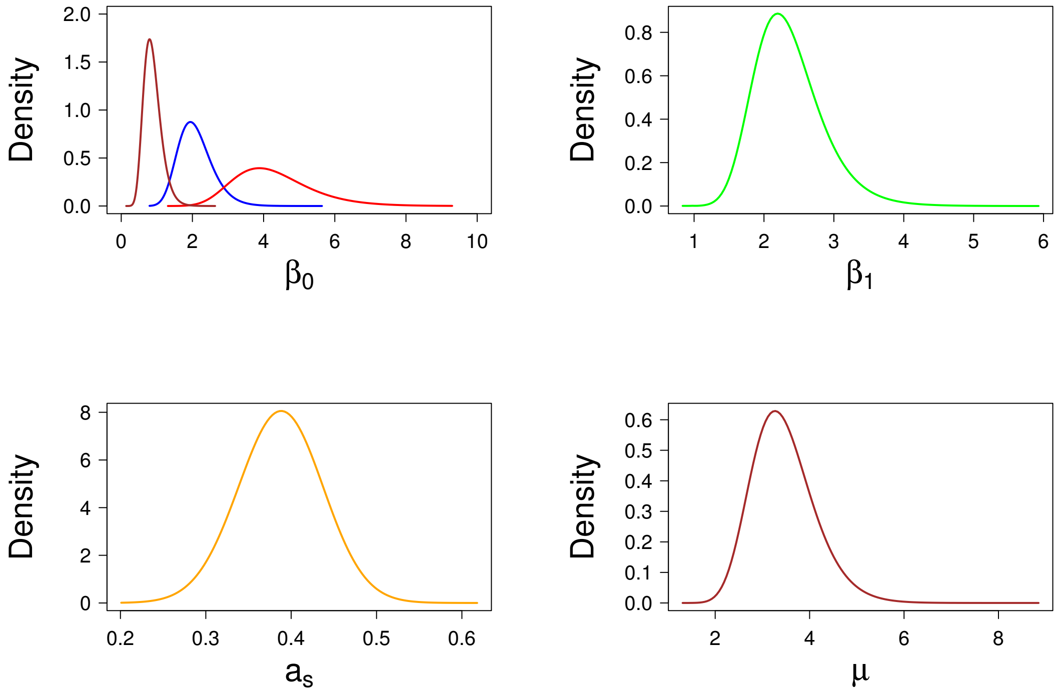

Marginal posterior densities for β_0, β_1, a_s and μ on the real scale using the informative prior for μ with mean=1.2 and sd=0.2. For β_0 brown is urban, blue is border and red is periurban.
