# Supplementary material for: Dynamics of the Force of Infection: Insights from Echinococcus multilocularis Infection in Foxes
Source: PLoS Negl Trop Dis. 2014 Mar 20;8(3):e2731. doi: 10.1371/journal.pntd.0002731 (PMC3961194; doi:10.1371/journal.pntd.0002731)
Supplement: Text S7 — Model prevalence estimates by habitat using model 1-P0. (PDF) [file pntd.0002731.s008.pdf]

Supporting Information Text S7

Modal prevalence estimates by habitat using Model 1-P_0

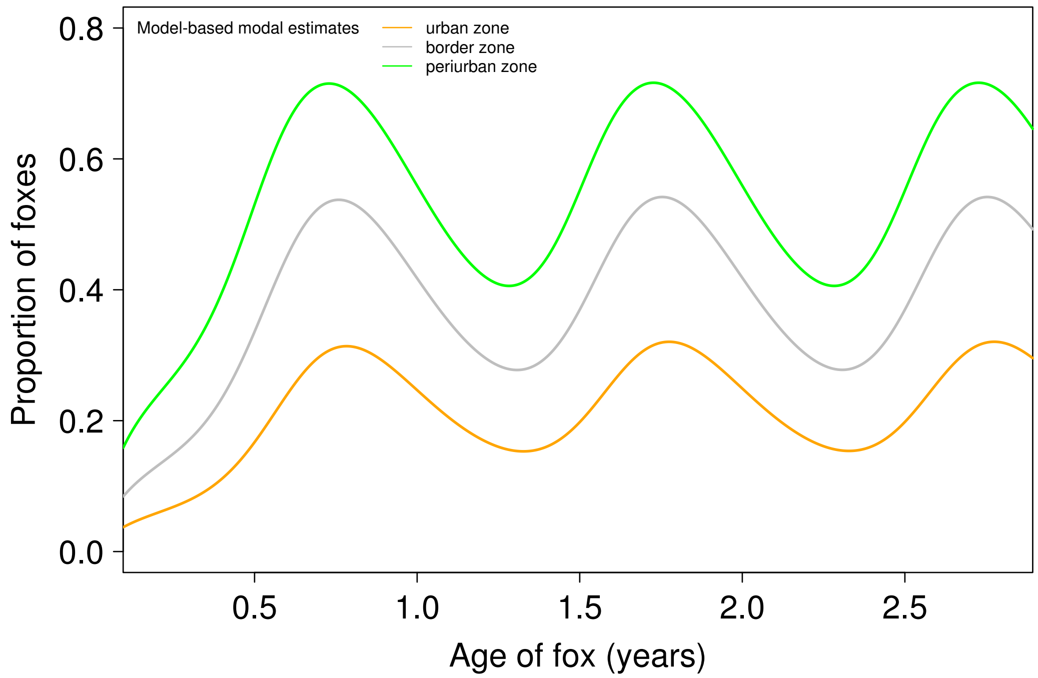

Model predicted prevalence using posterior mode estimates of all model parameters and using the informative prior for μ with mean=1.2 and sd=0.2.
